# Suspected Endothelial Pencil Graphite Deposition

**DOI:** 10.1155/2013/369374

**Published:** 2013-12-10

**Authors:** Adem Gül, Ertuğrul Can, Özlem Eşki Yücel, Leyla Niyaz, Halil İbrahim Akgün, Nurşen Arıtürk

**Affiliations:** Ondokuz Mayis University, Ophthalmology Department, Korfez Mah, Mehmet Akif Ersoy Bulvarı, No. 84/15, Atakum, 55100 Samsun, Turkey

## Abstract

A 14-year-old male patient had an ocular trauma with a pencil. Biomicroscopic examination revealed a broken part of pencil into the cornea. Foreign body removal and corneal wound closure were performed in the same day. After corneal repair, there was a grade 4+ anterior chamber reaction just like in preoperative examination. Dilated examination showed a very small piece broken tip of pencil on the upper nasal quadrant of the lens. A small and linear deposition was also seen on endothelial surface. Endothelial deposition and foreign body disappeared with intensive topical steroid treatment.

## 1. Introduction

Ocular trauma is not a rare condition which is mostly seen in children and it occupies an important area in ophthalmologic diseases that may result in some degree of vision loss [1]. Foreign bodies can be seen in anterior chamber, lens, iris, vitreous chamber, and retina after trauma. The most seen area of the foreign body is anterior chamber (15%), reported by Han et al. [2].

Although several etiologic factors have been identified, the most common ones are metal, stone, and wooden pieces. Pencil is a very rare etiologic agent [3]. As it is known, pencil is composed of wooden and lead parts. Graphite, which is the major constituent of pencil lead, has been reported to remain inert in the eye for a long time. When an injury to the globe with a pencil tip happens, there may be a deposition of the graphite that may remain inert or reactive, discussed by Honda and Asayama [4]. In this paper, a reactive foreign body will be presented and a deposition seen on endothelial surface will be discussed.

## 2. Case

**Figure 1:**
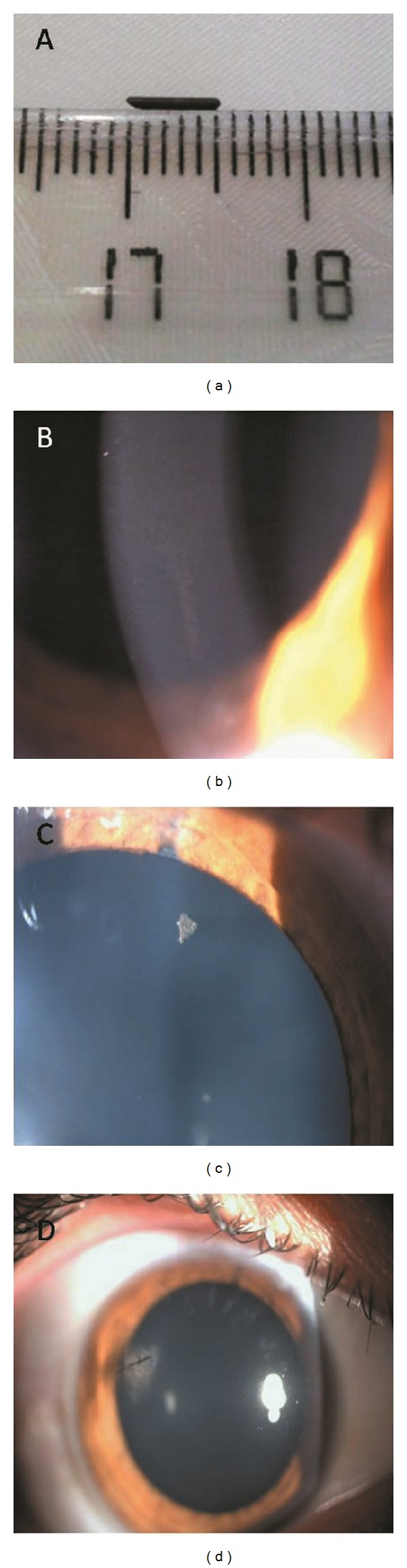
(a) Removed foreign body; (b) endothelial deposits; (c) graphite on the lens in first day; (d) corneal deposits were resolved.

A 14-year-old male patient was admitted to our hospital with a complaint of decreased vision in the right eye after an ocular trauma with a pencil. Patient was admitted to the hospital, nine hours after trauma. Visual acuity was finger count from two meters in the right eye and 10/10 in the left eye. Slit lamp examination revealed a corneal perforation and a part of pencil in the edges of the wound on the upper temporal region between 9 and 10 o'clock alignment in the right eye. There were no pathologic findings in the left eye. There was a grade 4+ anterior chamber reaction and a membrane formation within the perforated area. The patient was hospitalized and foreign body removal (Figure 1(a)) and corneal wound closure with nylon sutures were performed in the same day.

Written informed consent was obtained from the patient for publication of this case report and accompanying images.

Two days after corneal repair, visual acuity was 7/10 and intraocular pressure was 15 mm Hg. In the slit lamp examination, anterior chamber reaction was grade 4+ just like in preoperative examination. A small and linear deposition was also seen on endothelial surface (Figure 1(b)). When the affected eye was dilated for fundus examination, a very small piece of broken tip of the pencil was seen on upper nasal quadrant of the lens (Figure 1(c)). Treatment was started with moxifloxacin 400 mg oral tablet daily as systemic treatment and moxifloxacin drop eight times, cyclopentolate drop three times, and prednisolone drop twelve times daily as topical treatment.

Right anterior chamber reaction and membrane formation were resolved with the treatment in the following days (Figure 1(d)). Visual acuity was 10/10 in the third day postoperatively. Systemic treatment was stopped after a week and topical treatment was continued as moxifloxacin eight times and prednisolone twelve times daily.

## 3. Discussion

Graphite foreign bodies may be retained in the eye without causing any inflammation or damage to the intraocular structures but there is almost a possibility of progressive damage to intraocular structures. It may also lead to corneal microcysts, chronic anterior chamber reaction, keratitis, and even orbitocerebral abscesses. Due to its similar appearance with herpetic keratitis, these cases were usually treated with antiherpetic treatments as discussed elsewhere [2, 3, 5, 6].

There have been few reports of ocular graphite deposition in the literature [2, 5, 7–9]. Hamanaka et al. reported an 8-year-old boy who presented with an intraocular foreign body composed of graphite pencil lead. They performed corneal repair and lens extraction but two pieces of the pencil lead remained in the vitreous cavity following surgery, and 2 days later the patient developed endophthalmitis [5].

In another case, a growing vascular pigmented mass of the conjunctiva resembling a melanoma in a patient with a history of a pencil injury to the eye was surgically removed and histopathologically was found to be a graphite foreign body granuloma, discussed by Guy and Rao [7].

Han et al. reported a case of a retained graphite anterior chamber foreign body that was masquerading as stromal keratitis. They suspected herpetic stromal keratitis and treated it with antiviral and anti-inflammatory agents. Three months later, they incidentally identified a foreign body in the inferior angle of anterior chamber angle. It was surgically removed. The removed foreign body was a fragment of graphite and the patient subsequently disclosed a history of trauma with a mechanical pencil 12 years earlier [2].

In our case, there was a membrane around the pencil graphite and a grade 4+ anterior chamber reaction. After removal of the larger graphite, a persistent anterior chamber reaction and a deposition on endothelial surface were noted. Dilated examination showed a very small piece of the broken tip of the pencil on the upper nasal quadrant of the lens. According to the findings of endothelial deposition and small graphite on the lens, we thought that very small piece of graphite was releasing particles and these particles were deposited on the endothelial surface. Although histopathologic study is required to confirm diagnosis of graphite deposition on endothelial surface, it is not possible to take pathologic specimen from a living cornea. So, we named this deposition as “suspected graphite deposition.” We discussed with our colleagues performing anterior chamber irrigation to eliminate the very small graphite part, but in the first week, endothelial suspected graphite deposition and the graphite on the lens were resolved with the treatment of topical/systemic antibiotics and steroids. Although it cannot be proven with certainty, the anterior chamber inflammation was most likely due to the graphite since earlier reports have shown no inflammatory activity with similar kind of injuries.

We searched the literature with words “cornea, endothelium, graphite, pencil” but could not find any reports related to endothelial graphite deposition. So, this case may contribute to the literature as suspected graphite deposition on endothelial surface.
